# Pennsylvania Hospital

**Published:** 1859-05

**Authors:** 


PENNSYLVANIA HOSPITAL.—The vacancy of consulting physician at this institution, occasioned by the resignation of Dr. Pepper, has been filled by the appointment of Dr. John Forsyth Meigs. We congratulate the managers and medical staff upon the acquisition of a gentleman who enjoys so well-earned a reputation as an author, and who has exhibited such zeal in the pursuits of his profession. We are confident that Dr. Meigs will make good use of his observations, and that his instructions will be of great benefit to those who may avail themselves of them.

